# Supplementary material for: Heat adaptation of phage T7 under an extended genetic code
Source: Virus Evol. 2021 Dec 1;7(2):veab100. doi: 10.1093/ve/veab100 (PMC8923235; doi:10.1093/ve/veab100)
Supplement: veab100_Supp [file veab100_supp.zip › Sequencing_Analysis.zip!/Sequencing_Analysis/A2/evidence/JC_SIDE_2_48.html]

BRESEQ :: Evidence


| New junction evidence | | | | | | | | | | | |
| --- | --- | --- | --- | --- | --- | --- | --- | --- | --- | --- | --- |
|  | | seq id | position | reads (cov) | reads (cov) | score | skew | freq | annotation | gene | product |
| \* | ? | NC\_001604.1 | = 8253 | 443 (0.970) | 7 (0.020) | 7/584 | 20.4 | 1.4% | intergenic (–/–) | *–/–* | –/– |
|  | ? | NC\_001604.1 | 8503 = | 536 (1.210) |  |  |  |  | intergenic (–/–) | *–/–* | –/– |
| Rejected: Coverage evenness skew score above cutoff. | | | | | | | | | | | |
| Rejected: Frequency below/above cutoff threshold. | | | | | | | | | | | |

Only 100 of 548 total aligned reads displayed.  

`CGCCAGTACAAGCAACTGGGTGTATAGCTTGCTTAGAGAAAGATGACCGTTATCCACACACCTGTAACAAAGGAGCTAACGATATGACCGAACGTGAACAAGAGATGATCATTAAGTTGATAGACAATAATGAAGGTCGCCCAGATGATTTGAATGGCTGCGGTATTCTCTGCTCCAATGTCCCTTGCCACCTCTGCCCCGCAAATAACGATCAAAAGATAACCTTAGGTGAAATCCGAGCGATGGACCCACGTAAACCACATCTGAATAAACCTGAGGTAACTCCTACAGATGACCAGCCTTCCGCTGAGACAATCGAAGGTGTCACTAAGCCTTCCCACTACATGCTGTTTGACGACATTGAGGCTATCGAAGTGATTGCTCGTTCAATGACCGTTGAGCAGTTCAAGGGATACTGCTTCGGTAACATCTTAAAGTACAGACTACGTGCTGGTAAGAAGTCAGAGTTAGCGTACTTAGAGAAAGACCTAGCGAAAGCAGACTTCTATAAAGAACTCTTTGAGAAACATAAGGATAAATGTTATGCATAACTTCAAGTCAACCCCACCTGCCGACAGCCTATCTGATGACTTCACA  >  NC_001604.1/8206‑8802`  

                                                                                                                                                                                                                                                                                                         `|`                                                                                                                                                                                                                                                                                                             
                   `gtgtATAGCTTGCTTAGAGAAAGATGACCGTTATCCACACACCTGTAACAAAGGAGCTAACGATATGACCGAACGTGAACAAGAGATGATCATTAAGTTGATAGACAATAATGAAGGTCGCCCAGATGATTTGAATGGCTGCGGTATTCTCTGCTCCAATGTCCCTTGCCACCTCTGCCCCGCAAATAACGATCAAAAGATAACCTTAGGTGAAATCCGAGCGATGGACCCACGTAAACCACATCTGAATAAACCTGAGGTAACTCCTACAGATGACCAGCCTTCCGCTGAGACAATCGaa                                                                                                                                                                                                                                                                                       >  1:628045/1‑301 (MQ=255)`  
                      `tataGTCTGCTTAGAGAAAGATGACCGTTATCCACACACCTGTAACAAAGGAGCTAACGATATGACCGAAAGTGAACAAGAGATGATCATTAAGTTGATAGACAATAATGAAGGTCGCCCAGATGATTTGAATGGCTGCGGTATTCTCTGCTCCAATGTCCATTGCCACCTCTGCCCCGCAAATAACGATCAAAAGATAACCTTAGGTGAAATCCGAGCGATGGACCCACGTAAACCACATCTGAATAAACCTGAGGTAACTCCTACAGATGACCAGCCTTCCGCTGAGACAATCGAAGgt                                                                                                                                                                                                                                                                                    <  1:67016/301‑1 (MQ=255)`  
                       `ataGCATGCTTAGAGAAAGATTACCGTTATCCACACACCTGTAACAAAGGAGCTAACGATATGACCGAAAGTGAACAAGAGATGATCATTAAGTTGATATACAATAATGAAGGTCGCCCAGATGATTTGAATGGCTGCGGTATTCGCTGCTCCAATGTCCCTTGCCACCTCTGCCCCGCAAATAACGATCAAAAGATAACCTTAGGTGAAATCCGAGCGATGGAACCACGTAAACCACATCTGAATAAACCTGAGGTAACTCCTACAGATGACCAGCCTTCCGCTGAGACAATCGAAGgtg                                                                                                                                                                                                                                                                                   <  1:588079/301‑1 (MQ=255)`  
                               `cttAGAGAAAGATGACCGGTAACCACACACCTGTAACAAAGTAGCTAACTATATGACCGAACGTGAACAAGAGATGAACATTAAGGTGATAGACAATAATGAAGGTCGCCCAGATGATTTGAATGGCTGTGGTATTCTCTTCTCCAATGTCCCTTGCCACCTCTGCCCCGCAAATAACGATCAAAAGATAACCTTAGGTTAAATCCGAGCGATGGACCCACGTAAACCACATCTGAATAAACCTGAGGTAACTCCTACAGATGACCAGCCTTCCGCTGAGACAATCGAAGGTGTCACTAAg                                                                                                                                                                                                                                                                           <  1:198661/301‑1 (MQ=255)`  
                                 `tAGAGAAAGATGACCGTTATCCACACACCTGTAACAAAGGAGCTAACGATATGACCGAACGTGAACAAGAGATGATCATTAAGTTGATAGACAATAATGAAGGTCGCCCAGATGATTTGAATGGCTGCGGTATTCTCTGCTCCAATGTCCCTTGCCACTTCTGCCCCGCAAATAACGATCAAAAGATAACCTTAGGTGAAATCCGAGCGATGGACCCACGTAAACCACATCTGAATAAACCTGAGGTAACTCCTACAGATGACCAGCCTTCCGCTTAGACAATCGAAGGTGTCAATAAGcc                                                                                                                                                                                                                                                                         >  1:432991/1‑301 (MQ=255)`  
                                 `tAGAGAAAGATGACCGTTATCCACACACATGTAACAAAGGAGCTAACGATATGACCGAACGTGAACAAGAGATGATCATTAAGTTGATAGACAATAATGAAGGTCGCCCAGATGATTTGAATGGCTGCGGTATTCTCTGCTCAAATGTCCCTTGCCACCTCTGCCCCGCAAATAACGATCAAAAGATAACCTTAGGTGAAATCCGAGCGATGGACCCACGTAAACCACATCTGAATAAACCTGAGGTAACTCCTACAGATGACCAGCCTTCCGCTGAGACAATCGAAGGTGTCACTAAGcc                                                                                                                                                                                                                                                                         <  1:638833/301‑1 (MQ=255)`  
                                   `gagaAAGATGACCGTTATCCACACACCTGTAACAAAGGAGCTAACGATATGACCGAACGTGAACAAGAGATGATCATTAAGTTGATAGACAATAATGAAGGTCGCCCAGATGATTTGAATGGCTGCGGTATTCTCTGCTCCAATGTCACTTGCCACCTCTGCCCCGCAAATAACGATCAAAAGATAACCTTAGGTGAAATCCGAGCGATGGACCCACGTAAACCACATCTGAATAAACCTGAGGTAACTCCAACAGATGACCAGCCTTCCGCTGAGACAATCGAAGGTGTCACTAAGCCtt                                                                                                                                                                                                                                                                       >  1:503005/1‑301 (MQ=255)`  
                                     `gaAAGATGACCGTTATCCACACACCTGTAACAAAGGAGCTAACGATATGACCGAACGTGAACAAGAGATGATCATTAAGTTGATAGACAATAATGAAGGTCGCCCAGATGATTTGAATGGCTGCGGTATTCTCTGCTCCAATGTCCCTTGCCACCTCTGCCCCGCAAATAACGATCAAAAGATAACCTTAGGTGAAATCCGAGCGATGGACCCACGTAAACCACATCTGAATAAACCTGAGGTAACTCCTACAGATGACAAGCCTTCCGCTGAGACAATCGAAGTTTTCACTAAGCCTTcc                                                                                                                                                                                                                                                                     >  1:15872/1‑301 (MQ=255)`  
                                       `aaGATGACCGTTATCCACACACCTGTAACAAAGGAGCTAACGATATGACCGAACGTGAACAAGAGATGATCATTAAGTTGATAGACAATAATGAAGGTCGCCCAGAAGATTTGAATGGCTGCGGTATTCTCTGCTCCAATGTCCCTTGCCACCTCTGCCCCGCAAATAACGATCAAAAGATAACATTAGGTGAAATCCGAGCGATGGACCCACGTAAACCACATCTGAATAAACCTGAGGTAACTCCTACAGATGACCAGCCTTCCGCTGCGACAATCGAAGGTGTCACTAAGCCTTCCCa                                                                                                                                                                                                                                                                   >  1:791423/1‑301 (MQ=255)`  
                                        `gtgtataagagacagacacacCTGTAACAAAGGAGCTAACGATATGACCGAACGTGAACAAGATATGATCATTAAGTTGATAGACAATAATGAAGGTCGCCCAGATGATTTGAATGGCTGCGGTATTCTCTGCTCCAATGTCCCTTGCCACCTCTGCCCCGCAAATAACGATCAAAAGATAACCTTAGGTGAAATCCGAGCGATGGACCCACGTAAACCACATCTGAATAAACCTGAGGTAACTCCTACAGATGACCAGCCTTCCGCTGAGACAATCGAAGGTGTCACTAAGCCTTCCCAc                                                                                                                                                                                                                                                                  <  1:705204/286‑1 (MQ=255)`  
                                        `ataagagacagaTCCACACACCTGTAACAAAGGAGCTAACGATATGACCGAACGTGAACAAGAGATGATCATTAAGTTGATAGACAATAATGAAGGTCGCCCAGATGATTTGAATGGCTGCGGTATTCTCTGCTCCAATGTACCTTGCCACCTCTGCCACGCAAATAACGATCAAAAGATAACCTTAGGTGAAATCCGAGCGATGGACCCACGTAAACCACATCTGAATAAACCTGAGGTAACTCCTACAGATGACCAGCCTTCCGCTGAGACAATCGAAGGTGTCACTAAGCCTTCCCAc                                                                                                                                                                                                                                                                  <  1:775168/290‑1 (MQ=255)`  
                                        `aGATGACCGTTATCCACACACCTGTAACAAAGGAGCTAACGATATGACCGAACGTGAACAAGAGATGATCATTAAGTTGATAGACAATAATGAAGGTCGCCCAGATGATTTGAATGGCTGCGGTATTCTATGCTCCAATGTCCCTTGCAACCTCTGCCCCGCAAATAACGATCAAAAGATAACCTTAGGTGAAATCCGAGCGATGGACCCACGTAAACCACATCTGAATAAACCTGAGGTAACTCCTACAGATGACCAGCCTTCCGCTGAGACAATCGAAGGTGTCACTAAGCCTTACCAc                                                                                                                                                                                                                                                                  <  1:689810/301‑1 (MQ=255)`  
                                            `gACCGTTATCCACACACCTGTAACAAAGGAGCTAACGATATGACCGAACGTGAACAAGAGATGATCATTAAGTTGATAGACAATAATGAAGGTCGCCCAGATGATTTGAATGGCTGCGGTATTCTCTGCTCCAATGTCCCTTGCCACCTCTGCCCAGCAAATAACGATCAAAAGATAACCTTAGGTGAAATCCGAGCGATGGACCCACGTAAACCACATCTGAATAAACCTGAGGTAACTCCTACAGATGACCAGCCTTCCGCTGAGACAATCGAAGGTGTCACTAAGCCTTCCCACTACa                                                                                                                                                                                                                                                              >  1:303989/1‑301 (MQ=255)`  
                                             `aaagagacagacacacCTGTAACAAAGGAGCTAACGATATGACTGAACGTGAACAAGAGATGATCATTAAGTTGATAGACAATAATGAAGGTCGCCCAGATGATTTGAATGGCTGCGGTATTCTCTGCTCCAATGTCCCTTGCCACCTCTGCCCCGAAAATAACGATCAAAAGATAACCTTAGGTGAAATCCGAGCGATGGACCAACGTAAACCACATCTGAATAAACCTGAGGTAACTCCTACAGATGACCAGCCTTCCGCTGAGACAATCGAAGGTGTCACTAAGCCTTACCACTACAt                                                                                                                                                                                                                                                             <  1:59646/291‑1 (MQ=255)`  
                                               `gtataagagacagccTGTAACAAAGGAGCTAACGATATGACCGAACGTGAACAAGAGATGATCATTAAGTTGATAGACAATAATGAAGGTCGCCCAGATGATTTGAATGGCTGCGGTATTCTCTGCTCCAATGTCCCTTGCCACCTCTGCCCCGCAAATAACGATCAAAAGATAACCTTAGGTGAAATCCGAGCGATGGACCCACGTAAACCACATCTGAATAAACCTGAGGTAACTCCTACAGATGACCAGCCTTCCGCTGAGACAATCGAAGGTGTCACTAAGCCTTCACACTACATGc                                                                                                                                                                                                                                                           <  1:137760/288‑1 (MQ=255)`  
                                               `cGTTATCCACACACCTGTAACAAAGGAGCTAACGATATGACCGAACGTGAACAAGAGATGATCATTAAGTTGATAGACAATAATGAAGGTCGCCCAGATGATTTGAATGGCTGCGGTATTCTCTGCTCCAATGTCCCTTGCCACCTCTGCCCCGCAAATAACGATCAAAAGATAACCTTAGGTGAAATCCGAGCGATGGACCCACGTAAACCACATCTGAATAAACCTGAGGTAACTCCTACAGATGACCAGCCTTCCGCTGAGACAATCGAAGGTGTCACTAAGCCTTCCCACTACATGc                                                                                                                                                                                                                                                           <  1:321129/301‑1 (MQ=255)`  
                                                   `aTCCACACACCTGTAACAAAGGAGCTAACGATATGACCGAACGTGAACAAGAGATGATCATTAAGTTGATAGACAATAATGAAGGTCGCCCAGATGATTTGAATGGCTGCGGTATTCTCTGCTCCAATGTCCCTTGCCACCTCTGCCCCGCAAATAACGATCAAAAGATAACCTTAGGTGAAATCCGAGCGATGGACCCACGTAAACCACATCTGAATAAACCTGAGGTAACTCCTACAGATGACCAGCCTTCCGCTGAGACAATCGAAGGTGTCACTAAGCCTTCCCCCTACATGCTGtt                                                                                                                                                                                                                                                       >  1:369638/1‑301 (MQ=255)`  
                                                      `cacacaCCTGTAACAAAGGAGCTAACGATATGACCGAACGTGAACAAGAGATGATCATTAAGTTGATAGACAATAATGAAGGTCGCCCAGATGATTTGAATGGCTGCGGTATTCTCTGCTCCAATGTCCCTTGCCACCTCTGCTCCGCAAATAACGATCAAAAGATAACCTTAGGTGAAATCCGAGCGATGGACCCACGTAAACCACATCTGAATAAACCTGATGTAACTCCTACAGATGACCAGCCTTCCGCTGAGACAATCGAAGGTGTCACTAAGCCTTCCCACTACATGcctgtctc                                                                                                                                                                                                                                                    >  1:723420/1‑294 (MQ=255)`  
                                                          `cacCTGTAACAAAGGAGCTAACGATATGACCGAACGTGAACAAGAGATGATCATTAAGTTGATAGACAATAATGAAGGTCGCCCAGATGATTTGAATGGCTGCGGTATTCTCTGCTCCAATGTCCCTTGCCACCTCTGCCCCGCAAATAACGATCAAAAGATAACCTTAGGTGAAATCCGAGCGATGGACCCACGTAAACCACATCTGAATAAACCTGAGGTAACTCCTACAGATGACCAGCCTTCCGCTGAGACAATCGAAGGTGTCACTAAGCCTTCCCACTACATGCTGTTTgacgac                                                                                                                                                                                                                                                >  1:282794/1‑301 (MQ=255)`  
                                                            `ccTGTAACAAAGGAGCTAACGATATGACCGAACGTGAACAAGAGATGATCATTAAGTTGATAGACAATAATGAAGGTCGCCCAGATGATTTGAATGGCTGCGGTATTCTCTGCTCCAATGTCCCTTGCCACCTCTGCCCCGCAAATAACGATCAAAAGATAACCTTAGGTGAAATCCGAGCGATGGACCCACGTAAACCACATCTGAATAAACCTGAGGTAACTCCTACAGATGACCAGCCTTCCGCTGAGACAATCTAAGGTGTCACTAAGCCTTCCCACTACATGCTGTTTGACGACAt                                                                                                                                                                                                                                              >  1:118074/1‑301 (MQ=255)`  
                                                            `ccTGTAACAAAGGAGCTAACGATATGACCGAACGTGAACAAGAGATGATCATTAAGTTGATAGACAATAATGAAGGTCGCCCAGATGATTTGAATGGCTGCGGTATTCTCTGCTCCAATGTCCCTTGCCACCTCTGCCCCGCAAATAACGATCAAAAGATAACCTTAGGTGAAATCCGAGCGATGGACCCACGTAAACCACATCTGAATAAACCTGAGGTAACTCCTACAGATGACCAGCCTTCCGCTGAGACAATCGAAGGTGTCACTAAGCCTTCCCAcctgtctcttttacacatctc                                                                                                                                                                                                                                              >  1:100796/1‑281 (MQ=255)`  
                                                               `gTAACAAAGGAGCTAATGATATGACCGAACGTGAACAAGAGATGATCATTAAGTTGATAGACAATAATGAAGGTCGCCCAGATGATTTGAATGGCTGCGGTATTCTCTGCTCCAATGTCCCTTGCCACCTCTGCCCCGCAAATAACGATCAAAAGATAACCTTAGGTGAAATCCGAGCGATGGACCCACGTAAACCACATCTGAATAAACCTGAGGTAACTCCTACAGATGACCAGCCTTCCGCTGAGACAATCGAAGGTGTCACTAAGCATTCCCACTACATGCTGTTTGACGACATTGa                                                                                                                                                                                                                                           >  1:362196/1‑301 (MQ=255)`  
                                                                 `tgtgtataagagacagATATGACCGAACGTGAACAAGAGATGATCATTAAGTTGATAGACAATAATGAAGGTCGCCCAGATGATTTGAATGGCTGCGGTATTCTCTGCTCCAATGTCCCTTGCCACCTCTGCCCCGCAAATAACGATCAAAAGATAACCTTAGGTGAAATCCGAGCGATGGACCCACGTAAACCACATCTGAATAAACCTGAGGTAACTCCTACAGATGACCAGCCTTCCGCTGAGACAATCGAAGGTGTCACTAAGCCTTACCACTACATGCTGTTTGACGACATTGAgg                                                                                                                                                                                                                                         <  1:615715/286‑1 (MQ=255)`  
                                                                 `aagagacaggCTAACGATATGACCGAACGTGAACAAGAGATGATCATTAAGTTGATAGACAATAATGAAGGTCGCCCAGATGATTTGAATGGCTGCGGTATTCTCTGCTCCAATGTCCCTTGCCACCTCTGCCCAGCAAATAACGATCAAAAGATAACCTTAGGTGAAATCCGAGCGATGGACCCACGTAAACCACATCTGAATAAACCTGAGGTAACTCCTACAGATGACCAGCCTTCCGCTGAGACAATCGAAGGTGTCACTAAGCCTTCCCACTACATGCTGTTTGACGACATTGAgg                                                                                                                                                                                                                                         <  1:179832/292‑1 (MQ=255)`  
                                                                        `gAGCTAACGATATGACCGAACGTGAACAAGAGATGATCATTAAGTTGATAGACAATAATGAAGGTCGCCCAGATGATTTGAATGGCTGTGGTATTCTCTGCTCCAATGTCCCTTGCCACCTCTGCCCCGCAAATAACGATCAAAAGATAACCTTAGGTGAAATCCGAGCGATGGACCCACGTAAACCACATCTGAATAAACCTGAGGTAACTCCTACAGATGACCATCCTTCCGCTGAGACAATCGAAGGTGTCACTAAGCCTTCCCACTACATGCTGTTTGACGACATTGAGGCTATCGa                                                                                                                                                                                                                                  >  1:166101/1‑301 (MQ=255)`  
                                                                               `caatatGACCGAACGTGAACAAGAGATGATCATTAAGTTGATAGACAATAATGAAGGTCGCCCAGATGATTTGAATGGCTGCGGTATTCTCTGCTCCAATGTCCCTTGCCACCTCTGCCCCGCAAATAACGATCAAAAGATAACCTTAGGTGAAATCCGAGCGATGGACCCACGTAAACCACATCTGAATAAACCTGAGGTAACTCCTACAGATGACCATCCTTCCGCTGAGACAATCGAAGGTGTCACTAAGCCTTCCCACTACATGCTGTTTGACGACATTGAGGCTATCGAAGTGAtt                                                                                                                                                                                                                           >  1:262559/3‑301 (MQ=255)`  
                                                                                  `aatGACCGAACGTGAACAAGAGATGATCATTAAGTTGATAGACAATAATGAAGGTCGCCCAGATGATTTGAATGGCTGCGGTATTCTCTGCTCCAATGTCCCTTGCCACCTCTGCCCCGCAAATAACGATCAAAAGATAACCTTAGGTGAAATCCGAGCGATGGACCCACGTAAACCACATCTGAATAAACCTGAGGTAACTCATACAGATGACCAGCCTTCCGCTGAGACAATCGAAGGTGTCACTAAGCCTTCCCACTACATGCTGTTTGACGACATTGAGGCTATCGAAGTGATTGCt                                                                                                                                                                                                                        <  1:109093/300‑1 (MQ=255)`  
                                                                                  `aaggACTGAACGTGAACAAGAGATGATCATTAAGTTGATAGACAATAATGAAGGTCGCCCAGATGATTTGAATGGCTGCGGTATTCTCTGCTCCAATGTCCCTTGCCACCTCTGCCCCGCAAATAACGATCAAAAGATAACCTTAGGTGAAATCCGAGCGATGGACCCACGTAAACCACATCTGAATAAACCTGAGGTAACTCCTACAGATGACCAGCCTTCCGCTGAGACAATCGAAGGTGTCACTAAGCCTTCCCACTACATGCTGTTTGACGACATTGAGGCTATCGAAGTGATTGCt                                                                                                                                                                                                                        <  1:34779/298‑1 (MQ=255)`  
                                                                                          `aaCGTGAACAAGAGATGATCATTAAGTTGATAGACAATAATGAAGGTCGCCCAGATGATTTGAATGGCTGCGGTATTCTCTGCTCCAATGTCCCTTGCCACCTCTGCCCCGCAAATAACGATCAAAAGATAACCTTAGGTGAAATCCGAGCGATGGACCCACGTAAACCACATCTGAATAAACCTGAGGTAACTCCTACAGATGACCAGCCTTCCGCTGAGACAATCGAAGGTGTCACTAAGCCTTCCCACTACATGCTGTTTGACGACATTGAGGCTATCGAAGTGATTGCTCGTTCAAt                                                                                                                                                                                                                >  1:48571/1‑301 (MQ=255)`  
                                                                                             `gacagcAAGAGATGATCATTAAGTTGATAGACAATAATGAAGGTCGCCCAGATGATTTGAATGGCTGCGGTATTCTCTGCTCCAATGTCCCTTGCCACCTCTGCCCCGCAAATAACGATCAAAAGATAACCTTAGGTGAAATCCGAGCGATGGACCCACGTAAACCACATCTGAATAAACCTGAGGTAACTCCTACAGATGACCAGCCTTCCGCTGAGACAATCGAAGGTGTCACTAAGCCTTCCCACTACATGCTGTTTGAAGACATTGAGGCTATCGAAGTGATTGCTCGTTCAATGAc                                                                                                                                                                                                             <  1:13150/296‑1 (MQ=255)`  
                                                                                             `gTGAACAAGAGATGATCATTAAGTTGATAGACAATAATGAAGGTCGCCCAGATGATTTGAATGGCTGCTGTATTCTCTGCTCCAATGTCCCTTGCCACCTCTGCCCCGCAAATAACGATCACAAGATAACCTTAGGTGAAATCCGAGCGATGGACCCACGTAAACCACATCTGAATAACCCTGAGGTAACTCCTACAGATGACCAGCCTTCCGCTGAGACAATCGAAGGTGTCACTAAGCCTTCCCACTACATGCTGTTTGACGACATTGAGGCTATCGAAGTGATTGCTCGTTCAATGAc                                                                                                                                                                                                             >  1:822818/1‑301 (MQ=255)`  
                                                                                                  `cAAGAGATGATCATTAAGTTGATAGACAATAATGAAGGTCGCCCAGATGATTTGAATGGCTGCGGTATTCTCTGCTCCAATGTCCCTTGCAACCTCTGCCCCGCAAATAACGATCAAAAGATAACCTTAGGTGAAATCCGAGCGATGGACCCACGTAAACCACATCTGAATAAACCTGAGGTAACTCCTACAGATGACCAGCCTTCCGCTGAGACAATCGAAGGTGTCACTAAGCCTTCCCACTACATGCTGTTTGACGACATTGAGGCTATCGAAGTGATTGCTCGTTCAATGACCGTTg                                                                                                                                                                                                        >  1:122334/1‑301 (MQ=255)`  
                                                                                                     `gagacagtCATTAAGTTGATAGACAATAATGAAGGTCTCCCAGATGATTTGAATGGCTGCGGTATTCTCTGCTCCAATGTCCCTTGCCACCTCTGCCCCGCAAATAACGATCAAAAGATAACCTTAGGTGAAAGCCGAGCGATGGACCCACGTAAACCACATCTGAATAAACCTGAGGTAACTCCTACAGATGACCAGCCTTCCGCTGAGACAATCGAAGGTGTCACTAAGCCTTCCAACTACATGCTGTTTGACGACATTGAGGCTATCGAAGTGATTGCTCGTTAAATGACCGTTGAGc                                                                                                                                                                                                     <  1:380894/294‑1 (MQ=255)`  
                                                                                                     `gagaTGATCATTAAGTTGATATACAATAATGAAGGTCGCCCAGATGATTTGAATGGCTGCGGTATTCTCTGCTCCAATGTCCCTTGCCACCTCTGCCCCGCAAATAACGATCAAAAGATAACCTTAGGTGAAATCCGAGCGATGGACACACGTAAACCACATCTGAATAAACCTGAGGTAACTCCTACAGATGACCAGCCTTCCGCTGAGACAATCGAAGGTGTCACTAAGCCTTCCCACTACATGCTGTTTGACGACATTGAGGCTATCGAAGTGATTGCTCGTTCAATGACCGTTGAGc                                                                                                                                                                                                     <  1:497669/301‑1 (MQ=255)`  
                                                                                                     `gagaTGATCATTAAGTTGATAGACAATAATGAAGGTCGCCCAGATGATTTGAATGGCTGCGGTATTCTCTGCTCCAATGTCCCTTGCCACCTCTGCCCCGAAAATAACGATCAAAAGATAACCTTAGGTGAAATCCGAGCGATGGACCAACGTAAACCACATCTGAATAAACCTGAGGTAACTCCTACAGATGACCAGCCTTCCGCTGAGACAATCGAAGGTGTCACTAAGCCTTCCCACTACATGCTGTTTGACGACATTGAGGCTATCGAAGTGATTGCTCGTTCAATGACCGTTGAGc                                                                                                                                                                                                     <  1:531423/301‑1 (MQ=255)`  
                                                                                                       `gatgatCATTAAGTTGATAGACAATAATGAAGGTCGCCCAGATGATTTGAATGGCTGTGGTATTCTCTGCTCCAATGTCCCTTGCCACCTCTGCCCCGCAAATAACGATCAAAAGATAACCTTAGGTGAAATCCGAGCGATGGACCCACGTAAACCACATCTGAATAAACCTGAGGTAACTCCTACAGATGACCAGCCTTCCGCTGAGACAATCGAAGGTGTCACTAAGCCTTCCCACTACATGCTGTTTGACGACATTGAGGCTATCGAAGTGATTGCTCGTTCAATGACCGTTGAGCAg                                                                                                                                                                                                   <  1:17616/301‑1 (MQ=255)`  
                                                                                                          `gatCATTAAGTTGATAGACAATAATGAAGGTCGCCCAGATGATTTGAATGGCTGCGGTATTCTCTGCTCCAATGTCCCTTGCCACCTCTGCCCCGCAAATAACGATCAAAAGATAACCTTAGGTGAAATCCGAGCGATGTACCCACGTAAACCACATCTGAATAAACCTGAGGTAACTCCTACAGATGACCAGCCTTCCGCTGAGACAATCGAAGGTGTCACTAAGCCTTCCCACTACATGCTGTTTGACGACATTGAGGCTATCGAAGTGATTGCTCGTTCAATGACCGTTGAGCAGTTc                                                                                                                                                                                                <  1:295501/301‑1 (MQ=255)`  
                                                                                                               `ttAAGTTGATAGACAATAATGAAGGTCGCCCAGATGATTTGAATGGCTGCGGTATTCTCTGCTCCAATGTCCCTTGCCACCTCTGCCCCGCAAATAACGATCAAAAGATAACCTTAGGTGAAATCCGAGCGATGGACCCACGTAAACCACATCTGAATAAACCTGAGGTAACTCCTACAGATGACCAGCCTTCCGCTGAGACAATCGAAGGTGTCACTAAGCCTTCCCACTACATGCTGTTTGACGACATTGAGGCTATCGAAGTTATTGCTCGTTCAATGACCGTTGAGCAGTTCAAggg                                                                                                                                                                                           <  1:387076/301‑1 (MQ=255)`  
                                                                                                               `ttAAGTTGATAGACAATAATGAAGGTAGCCCAGATGATTTGAATGGCTGCGGTATGCTCTGCTCCAATGTCCCTCGCCACCGCTGCCCCGCAAATAACGATCAAAAGATAACCTTAGGTGAAATCCGATCGATGGACCCACGTAAACCACATCTGAATAAACCTGAGGTAACTCCTACAGATGACCAGCCTTCCGCTGAGACAATCGAAGGTGTCACTAAGCCTTCCCACTACATGCGGTTTGACGACATTGAGGCTATCGAAGGTATTGCTCGTTCAATGACCGTTGAGCAGTTCAAggg                                                                                                                                                                                           <  1:491088/301‑1 (MQ=255)`  
                                                                                                               `gtataagagacagcAATAATGAAGGGCGCCCAGATGATTTGAATGGCTGCGGTATTCTCTGCTCCAATGTCCCTTGCCACCTCTGCCCCGCAAATAACGATCAAAAGATAACCTTAGGTGAAATCCGAGCGATGGACCCACGTAAACCACATCTGAATAAACATGAGGTAACTCCTACAGATGACCAGCCGTCCGCTGAGACAATCGAAGGTGTCACTAAGCCTTCACACTACATGCTGTTTGACGACATTGAGGCTATCGAAGTTATTGCTCGTTCAATGACCGTTGAGCAGTTCAAggg                                                                                                                                                                                           <  1:312735/288‑1 (MQ=255)`  
                                                                                                                           `aCAATAATGAAGGTCGCCCAGATGATTTGAATGGCTGCGGTATTCTCTGCTCCAATGTCCCTTGCCACCTCTGCCCCGCAAATAACGATCAAAAGATAACCTTAGGTGAAATCCGAGCGATGGACCCACGTAAACCACATCTGAATAAACCTGAGGTAACTCCTACAGATGACCAGCCTTCCGCTGAGACAATCGAAGGTGTCACTAAGCCTTCCCACTACATGCTGTTTGACGACATTGAGGCTATCGAAGTGATTGCTCGTTCAATGACCGTTGAGCAGTTCAAGGGATACTGCTTCgg                                                                                                                                                                               <  1:156489/301‑1 (MQ=255)`  
                                                                                                                             `aataatGAAGGTCGCCCAGATGATTTGAATGGCTGCGGTATTCTCTGCTCCAATGTCCCTTGCCACCTCTGCCCCGCAAATAACGATCAAAAGATAACCTTAGGTGAAATCCGAGCGATGTACCCACGTAAACCACATCTGAATAAACCTGAGGTAACTCCTACAGATGACCAGCCTTCCGCTGAGACAATCGAAGGTGTCACTAAGCCTTCCCACTACATGCTGTTTGACGACATTGAGGCTATCGAAGTGATTGCTAGTTCAATGACCGTTGAGCAGTTCAAGGGATACTGCTTCGGTa                                                                                                                                                                             <  1:35374/301‑1 (MQ=255)`  
                                                                                                                              `cgtgggctcggagatgtgtataagagacaggCTGCGGTATTCTCTGCTCCAATGTCCCTTGCCACCTCTGCCCCGCAAATAACGATCAAAAGATAACCTTAGGTGAAATCCGAGCGATGGACCCACGTAAACCACATCTGAATAAACCTGAGGTAACTCCTACAGATGACCAGCCTTCCGCTGAGACAATCGAAGGTGTCACTAAGCCTTCCCACTACATGCTGTTTGACGACATTGAGGCTATCGAAGTGATTGCTCGTTCAATGACCGTTGAGCAGTTCAAGGGATACTGCTTCGGTaa                                                                                                                                                                            <  1:679033/272‑1 (MQ=255)`  
                                                                                                                              `ataatGAAGGTCGCCCAGATGATTTGACTGGCTGCGGTATTCTCTGCTCCAATGTCCCTTGCCACCTCTGCCCCGCAAATAACGATCAAAAGATAACCTTAGGTGAAATCCGAGCGATGGACCCACGTAAACCACATCTGAATAAACCTGAGGTAACTCCTACAGATGACCAGCCTTCCGCTGAGACAATCGAAGGTGTCACTAAGCCTTCCCACTACATGCTGTTTAACGACATTGAGGCTATCGAAGTGATTGCTCGTTCAATGACCGTTTAGCATTTCAACGGATAcctgtctcttat                                                                                                                                                                            >  1:414601/1‑290 (MQ=255)`  
                                                                                                                              `ataatGAAGGTCGCCCAGATGATTTGAATGGCTGCGGTATTCTCTGCTCCAATGTCCCTTGCCACCTCTGCCCCGCAAATAACGATCAAAAGATAACCTTAGGTGAAATCCGAGCGATGGACCCACGTAAACCACATCTGAATAAACCTGAGGTAACTCCTACAGATGACCAGCCTTCCGCTGAGACAATCGAAGGTGTCACTAAGCCTTCCCACTACATGCTGTTTGACGACATTGAGGCTATCGAAGTGATTGCTCGTTCAATGACCGTTGAGCAGTTCAAGGGATACTGCTTCGGTaa                                                                                                                                                                            >  1:337891/1‑301 (MQ=255)`  
                                                                                                                              `ataatGAAGGTCGCCCAGATGATTTGAATGGCTGCGGTATTCTCTGCTCCAATGTCCCTTGCCACCTCTGCCCCGCAAATAACGATCAAAAGATAACCTTAGGTGAAATCCGAGCGATGGACCCACGTAAACCACATCTGAATAAACCTGAGGTAACTCCTACAGATGACCAGCCTTCCGCTGAGACAATCGAAGGTGTCACTAAGCCTTCCCACTACATGCTGTTTGACGACATTGAGGCTATCGAAGTGATTGCTCGTTCAATGACCGTTGAGCAGTTCAAGGGATACTGCTTCAGTaa                                                                                                                                                                            <  1:761065/301‑1 (MQ=255)`  
                                                                                                                                  `tGAAGGTCGCCCAGATGATTTGAATGGCTGCGGTATTCTCTGCTCCAATGTCCCTTGTCACCTCTGCCCCGCAAATAACGATCAAAAGATAACCTTATGTGAAATCCGAGCGATGGACCCACGTAAACCACATCTGAATAAACCTGAGGTAACTCCTACAGATGACCAGCCTTCTGCTGAGACAATCGAAGGTGTCACTAAGCCTTCCCACTACATGCTGTTTGACGACATTGAGGCTATCGAAGTGATTGCTCGTTCAATGACCGTTGAGCAGTTCAAGGGATACTGCTTCGGTAACATc                                                                                                                                                                        <  1:285981/301‑1 (MQ=255)`  
                                                                                                                                             `cgtgggctcggagatgtgtataagagacaggCTCCAATGTCCCTTGCCACCTCTGCCCCGCAAATAACTATCAAAAGATAACCTTAGGTGAAATCCGAGCGATGGACCCACGTAAACCACATCTGAATAAACCTGAGGTAACTCCTACAGATGACCAGCCTTCCGCTGAGACAATCGAAGGTGTCACTAAGCCTTCCAACTACATGCTGTTTGACGACATTGAGGCTATCGAAGTGATTGCTCGTTCAATGACCGTTGAGCAGTTCAAGGGATACTGCTTCGGTAACATCTTAAAGTACAg                                                                                                                                                             <  1:247009/271‑1 (MQ=255)`  
                                                                                                                                                `atgatTTGAATGGCTGCGGTATTCTCTGCTCCAATGTCCCTTGCCACCTCTGCCCCGCAAATAACGATCAAAAGATAACCTTAGGTGAAATCCGAGCGATGGACCCACGTAAACCACATCTGAATAAACCTGAGGTAACTCCTACAGATGACCAGCCTTCCGCTGAGACAATCTAAGGTGTCACTAAGCCTTCCCACTACATGCTGTTTGACGACATTGAGGCTATCGAAGTGATTGCTCGTTCAATGACCGTTGAGCAGTTCAAGGGATACTGCTTCGGTAACATCTTAAAGTACAGACt                                                                                                                                                          <  1:93724/301‑1 (MQ=255)`  
                                                                                                                                                `atgatTTGAATGGCTGCGGTATTCTCTGCTCCAATGTCCCTTGCCACCTATGCACCGCAAATAACGATCAAAAGATAACCGTAGGTGAAATCCGAGCGATGGACCCACGTAAACCACATCTGAATAAACCTGAGGTAACTCCTACAGATGACCAGCCTTCCGCTGAGACAATCGAAGGTGTCACTAAGCCTTCCCACTACATGCTGTTTGACGACATTGAGGCTATAGAAGTGATTGCTCGTTAAATGACCGTTGAGCAGTTCAAGGGATACTGCTTCGGTAACATCTTAAAGTACAGACt                                                                                                                                                          <  1:275050/301‑1 (MQ=255)`  
                                                                                                                                                     `ttGAATGGCTGCGGTATTCTCTGCTCCAATGTCCCTTGCCACCTCTGCCCCGCAAATAACGATAAAAAGATAACCTTAGGTGAAATCAGAGCGATGGACCAACGTATACCACATCTGAATAAACCTGAGGTAACTCCTACAGATGACCAGCCTTCCGCTGAGACAATCGAAGGTGTCACTAAGCCTTCCCACTACATGCTGTTTGACGACATTGAGGCTATCGAAGTGATTGCTCGTTCAATGACCGTTGAGCAGTTCAAGGGATACTGCTTCGGTAACATCTTAAAGTACAGACTACGTg                                                                                                                                                     <  1:474041/301‑1 (MQ=255)`  
                                                                                                                                                           `ggCTGCGGTATTCTCTGCTCCAATGTCCCTTGCCACCTCTGCCCCGCAAATAACGATCAAAAGATAACCTTAGGTGAAATCCGAGCGATGGACCCACGTAAACCACATCTGAATAAACCTGAGGTAACTCCTACAGATGACCAGCCTTCCGCTGAGACAATCGAAGGTGTCACTAAGCCTTCCCACTACATGCTGTTTGACGACATTGAGGCTATCGAAGTGATTGCTCGTTCAATGACCGTTGAGCAGTTCAAGGGATATTGCTTCGGTAACATCTTAAAGTACAGAcctgtctcttata                                                                                                                                               >  1:598675/1‑289 (MQ=255)`  
                                                                                                                                                                `gggTATTCTCTGCTCCAATGTCCCTTGCCACCTCTGCCCCGCAAATAACGATCAAAAGATAACCTTAGGTGAAATCCGAGCGATGGACCAACGTAAACCACATCTGAATAAACCTGAGGTAACTCCTACAGATGACCAGCATTCCGCTGAGACAATCGAAGGTGTCACTAAGCCTTCCCACTACATGCTGTTTGACAACATTGAGGCTATCGAAGTGATTGCTCGTTCAATGACCGTTGAGAAGTTCAAGGGATACTGCTTCGGTAACATCTTAAAGTACAGACTACGTGCTGGTaagaag                                                                                                                                          <  1:649346/300‑1 (MQ=255)`  
                                                                                                                                                                `cgtgggctcggagatgtgtataagagacagccTCTGCCCCGCAAATAACGATCAAAAGATAACCTTAGGTGAAATCCGAGCGATTGACCCACGTAAACCACATCTGAATAAACCTGAGGTAACTCCTACAGATGACCAGCCTTCCGCTGAGACAATCGAAGGTGTCACTAAGCCTTCCCACTACATGCTGTTTGACGACATTGAGGCTATCGAAGTGATTGCTCGTTCAATGACCGTTGAGCAGTTCAAGGGATACTGCTTCGGTAACATCTTAAAGTACAGACTACGTGCTGGTaagaag                                                                                                                                          <  1:524718/271‑1 (MQ=255)`  
                                                                                                                                                                `cGGTATTCTCTGCTCCAATGTCCCTTGCCACCTCTGCCCCGCAAATAATGATCAAAAGATAACCTTAGGTGAAATCCGAGCGATGGACCCACGTAAACCACATCTGAATAAACCTGAGGTAACTCCTACAGATGACCAGCCTTCCGCTGAGACAATCGAAGGTGTCACTAAGCCTTCCCACTACATGCTGTTTGACGACATTGAGGCTATCGAAGTGATTGCTCGTTCAATGACCGTTGAGCAGTTCAATGGATACTGCTTCGGTAACATCTTAAAGTACAGACTACGTGcctgtctctta                                                                                                                                          >  1:696320/1‑291 (MQ=255)`  
                                                                                                                                                                `cGGTATTCTCTGCTCCAATGTCCCTTGCCACCTCTGCCCCGCAAATAACGATCAAAAGATAACCTTAGGTGAAATCCGAGCGATGGACCCACGTAAACCACATCTGAATAAACCTGAGGTAACTCCTACAGATGACCAGCCTTCCGCTGAGACAATCGAAGGTGTCACTAAGCCTTCCCACTACATGCTGTTTGACGACATTGAGGCTATCGAAGTGATTGCTCGTTCAATGACCGTTGAGCAGTTCAAGGGATACTGCTTCGGTAACATCTTAAAGTACAGACTACGTGCTGGTaagaag                                                                                                                                          <  1:185796/301‑1 (MQ=255)`  
                                                                                                                                                                `agatgtgtataagagacaggTCCCTTGCCACCTCTGCCCCGCAAATAACGATCAAAAGATAACCTTAGGTGAAATCCGAGCGATGGACCCACGTAAACCACATCTGAATAAACCTGAGGTAACTCCTACAGATGACCAGCCTTCCGCTGAGACAATCGAAGGTGTCACTAAGCCTTCCCACTACATGCTGTTTGACGACATTGAGGCTATCGAAGTGATTGCTAGTTCAATGACCGTTGAGCAGTTCAAGGGATACTGCTTCGGTAACATCTTAAAGTACAGACTACGTGCTGGTaagaag                                                                                                                                          <  1:341121/282‑1 (MQ=255)`  
                                                                                                                                                                    `aTTCTCTGCTCCAATGTCCCTTGCCACCTCTGCCCCGCAAATAACGATCAAAAGATAACCTTAGGTGAAATCCGAGCGATGGACCCACGTAAACCACATCTGAATAAACCTGAGGTAACTCCTACAGATGACCAGCCTTCCGCTGAGACAATCGAAGGTGTCACTAAGCCTTCCCACTACATGCTGTTTGACGACATTGAGGCTATCGAAGTGATTGCTCGTTCAATGACCGTTGAGCAGTTCAAGGGATACTGCTTCGGTAACATCTTAAAGTACAGACTACGTGCTGGTAAGCAGTCag                                                                                                                                      >  1:563324/1‑301 (MQ=255)`  
                                                                                                                                                                              `ccAATGTCCCTTGCCACCTCTGCCCCGCAAATAACGATCAAAAGATAACCTTAGGTGAAATCCGAGCGATGGACCCACGTAAACCACATCTGAATAAACCTGAGGTAACTCCTACAGATGACCATCCTTCCGCTGAGACAATCGAAGGTGTCACTAAGCCTTCCCACTACATGCTGTTTGACGACATTGAGGCTATCGAAGTGATTGCTCGTTCAATGACCGTTGAGCAGTTCAAGGGATACTGCTTCGGTAACATCTTAAAGTACAGACTACGTGCTGGTAAGAAGTCAGAGTTAGCGTa                                                                                                                            >  1:581174/1‑301 (MQ=255)`  
                                                                                                                                                                                   `gTCCCTTGCCACCTCTGCCCCGCAAATAACGATCAAAAGATAACCTTAGGTGAAATCCGAGCGATGGACCCACGTAAACCACATCTGAATAAACCTGAGGTAACTCCTACAGATGACCAGCCTTCCGCTGAGACAATCGAAGGTGTCACTAAGCCTTCCCACTACATGCTGTTTGACGACATTGAGGCTATCGAAGTGATTGCTCGTTCAATGACCGTTGAGAAGTTCAATGGATACTGCTTCGGTAACATCTTAAAGTACAGACTACGTGCTgctgtcccttatacacatctccgagccc                                                                                                                       >  1:795806/1‑274 (MQ=255)`  
                                                                                                                                                                                         `tGCCACCTCTGCCCCGCAAATAATGATCAAAAGATAACCTTAGGTGAAATCCGAGCTATGGACCCACGTAAACCACATCTGAATAAACTTGAGGTAACTCCTACAGATGACCAGCCTTCCGCTGAGACAATCGAAGGTGTCACTAAGCCTTCCCACTACATGCTGTTTGACGACATTGAGGCTATCGAAGTGATTGCTCGTTCAATGACCGTTGAGCAGTTCAAGGGATACTGCTTCGGTAACATCTTAAAGTACAGACTACGTGCTGGTAAGAAGTCagagctgtctcttatacacatct                                                                                                                 >  1:224425/1‑282 (MQ=255)`  
                                                                                                                                                                                         `tGCCACCTCTGCCCCGCAAATAACGATCAAAAGATAACCTTAGGTGAAATCCGAGCGATGGACCCACGTAAACCACATCTGAATAAACCTGAGGTAACTCCTACAGATGACCAGCCTTCCGCTGAGACAATCGAAGGTGTCACTAAGCCTTCCCACTACATGCTGTTTGACGACATTGAGGCTATCGAAGTGATTGCTCGTTCAATGACCGTTGAGCAGTTCAAGGGATACTGCTTCGGTAACATCTTAAAGTACCGACTACGTGCTGGTAAGAAGTCAGAGttctgtctcttatacacat                                                                                                                 >  1:216683/1‑284 (MQ=255)`  
                                                                                                                                                                                           `ccACCTCTGCCCCGCAAATAACGATCAAAAGATAACCTTAGGTGAAATCCGAGCGATGGACCCACGTAAACCACATCTGAATAAACCTGAGGTAACTCCTACAGATGACCAGCCTTCCGCTGAGACAATCGAAGGTGTCACTAAGCCTTCCCACTACATGCTGTTTGACGACATTGAGGCTATCGAAGTGATTGCTCGTTCAATGACCGTTGAGCAGTTCAAGGGATACTGCTTCGGTAACATCTTAAAGTACAGACTACGTGCTGGTAAGAAGTCAGAGTTAGCGTACTTAGagacagac                                                                                                               >  1:192469/1‑301 (MQ=255)`  
                                                                                                                                                                                             `tgggctcggagatgtgtataagagacaggATAACCTTAGGTGAAATCCGAGCGATGGACCCACGTAAACCACATCTGAATAAACCTGAGGTAACTCCTACAGATGACCAGCCTTCAGCTGAGACAATCGAAGGTGTCACTAAGCCTTCCCACTACATGCTGTTCGACGACATTGAGGCTATCGAAGTGATTGCTCGTTAAATGACCGTTGAGCAGTTCAAGGGATACTGCTTCGGTAACATCTTAAAGTACAGACTACGTGCTGGTAAGAAGTCAGAGTTAGCGTACTTAGAGAAAGACCt                                                                                                             <  1:329050/273‑1 (MQ=255)`  
                                                                                                                                                                                                      `ccGCAAATAACGATCAAAAGATAACCTTAGGTGAAATCCGAGCGATGGACCCACGTAAACCACATCTGAATAAACCTGAGGTAACTCATACAGATGACCAGCCTTCCGCTGAGACAATCGAAGGTGTCACTAAGCCTTCCCACTACATGCTGTTTGACGACATTGAGGCTATCGAAGTGATTGCTCGTTCAATGACAGTTGAGCAGTTCAAGGGATACTGCTTCGGTAACATCTTAAAGTACAGACTACGTGCTGGTAAGAAGTCAGAGTTAGCGTACTTAGAGAAAGACCTAGCAAAAGc                                                                                                    <  1:783609/301‑1 (MQ=255)`  
                                                                                                                                                                                                             `cggagatgtggataagagacagggTGAAAACCGAGCGATGGACCCACGTAAACCACATCTGAATAAACCTGAGGTAACTCCTACAGATGACCAGCCTTCCGCTGAGACAATCGAAGGTGTCACTAAGCCTTCCCACTACATGCTGTTTGACGACATTGAGGCTATCGAAGTGATTGCTCGTTCAATGACCGTTGAGCAGTTCAAGGGATACTGCTTCGGTAACATCTTAAAGTACAGACTACGTGCTGGTAAGAAGTCAGAGTTAGCGTACTTAGAGAAAGACCTAGCGAAAGCAGACTTc                                                                                             <  1:742052/279‑1 (MQ=255)`  
                                                                                                                                                                                                                  `tataagagacagccTTAGGTGAAATCCGAGCGATGGACCCACGTAAACCACATCTGAATAAACCTGAGGTAACTCCTACAGATGACCAGCCTTCCGCTGAGACAATCGAAGGTGTCACTAAGCCTTCCCACTACATGCTGTTTGACGACATTGAGGCTATCGAAGTGATTGCTCGTTCAATGACCGTTGAGCAGTTCAAGGGATACTGCTTCGGTAACATCTTAAAGTACAGACTACGTGCTGGTAAGAAGTCAGAGTTAGCGTACTTAGAGAAAGACCTAGCGAAAGCAGACTTCTATaa                                                                                        <  1:550972/289‑1 (MQ=255)`  
                                                                                                                                                                                                                  `gtgggtataagggacagggTGAAAGCCGAGCGATGGACCCACGTAAACCACATCTGAATAAACCTGAGGTAACTCCTACAGATGACCAGCCTTCCGCTGAGACAATCGAAGGTGTCACTAAGCCTTCCCACTACATGCTGGTTGACGACATTGAGGCTATCGAAGTGATTGCTCGTTCAATGACCGTTGAGCAGTTCAAGGGATACTGCTTCGGTAACATCGTAAAGTACAGACTACGTGCTGGTAAGAAGTCAGAGTTAGCGGACTTAGAGAAAGACCTAGCGAAAGCAGACTTCTATaa                                                                                        <  1:520073/284‑1 (MQ=255)`  
                                                                                                                                                                                                                  `aTCAAAAGATAACCTTAGGTGAAATCCGAGCGATGGACCCACGTAAACCACATCTGAATAAACCTGAGGTAACTCCTACAGATGACCAGCCTTCCGCTGAGACAATCGAAGGTGTCACTAAGCCTTCCCACTACATGCTGTTTGACGACATTGAGGCTATCGAAGTGATTGCTCGTTCAATGACCGTTGAGCAGTTCAAGGGATACTGCTTCGGTAACATCTTAAAGTACAGACTACGTGCTGGTAAGAAGTCAGAGTTAGCGTACTTAGATAAAGACCTAGCGAAAGCAGACTTCTATaa                                                                                        <  1:209357/301‑1 (MQ=255)`  
                                                                                                                                                                                                                              `ccTTAGGTGAAATCCGAGCGATGGACCCATGTAAACCACATCTGAATAAACCTGAGGTAACTCCTACAGATGACCAGCCTTCCGCTGAGACAATCGAAGGTGTAACTAAGCCTTCCCACTACATGCTGTTTGACGACATTGAGGCTATCGAAGTGATTGCTCGTTCAATGACCGTTGAGCAGTTCAAGGGATACTGCTTCGGTAACATCTTAAAGTACAGACTACGTGCTGGTAAGAAGTCAGAGTTAGCGTACTTAGAGAAAGACCTAGCGAAAGCAGACTTCTATaactgtctcttata                                                                            >  1:658921/1‑289 (MQ=255)`  
                                                                                                                                                                                                                                         `aTCCGAGCGATGGACCAACGTAAACCACATCTGAATAAACCTGAGGTAACTCCTACAGATGACCAGCCTTCCGCTGAGACAATCGAAGGTGTCACTAAGCCTTCCCACTACATGCTGTTTGACGACATTGAGGCTATCGAAGTGATTGCTCGTTCAATGACCGTTGAGCAGTTCAAGGGATACTGCTTCGGTAACATCTTAAAGTACAGACTACGTGCTGGTAAGAAGTCAGAGTTAGCGTACTTAGAGAAAGACCTAGCGAAAGCAGACTTCTATAAAGAACTCTTTGAGAAACATAAgg                                                                 >  1:803882/1‑301 (MQ=255)`  
                                                                                                                                                                                                                                           `tgtataagagacagcACGTAAACCACATCTGAATAAACCTGAGGTAACTCCTACAGATGACCAGCCTTCCGCTGAGACAATCGAAGGTGTCACTAAGCCTTCCCACTACATGCTGTTTGACGACATTGAGGCTATCGAAGTGATTGCTCGTTCAATGACCGTTGAGCAGTTCAAGGGATACTGCTTCGGTAACATCTTAAAGTACAGACTACGTGCTGGTAAGAAGTCAGAGTTAGCGTACTTAGAGAAAGACCTAGCGAAAGCAGACTTCTATAAAGAACTCTTTGAGAAACATAAGGAt                                                               <  1:755724/287‑1 (MQ=255)`  
                                                                                                                                                                                                                                                `cGATGGACCAACGTAAACCACATCTGAATAAACCTGAGGTAACTCCTACAGATGACCAGCCTTCCGCTGAGACAATCGAAGGTGTCACTAAGCCTTCCCACTACATGCTGTTTGACGACATTGAGGCTATCGAAGTGATTGCTCGTTCAATGACCGTCGAGCAGTTCAAGGGATACTGCTTCGGTAACATCTTAAAGTACAGACTACGTGCTGGTAAGAAGTCAGAGTTAGCGTACTTAGAGAAAGACCTAGCGAAAGCAGACTTCTATAAAGAACTCTTTGAGAAACATAAGGATAAATg                                                          <  1:373462/301‑1 (MQ=255)`  
                                                                                                                                                                                                                                                 `gATGTACCCACGTAAACCACATCTGAATAAACCTGAGGTAACTCCTACAGATGACCAGCCTTCCGCTGAGACAATCGAAGGTGTCACTAAGCCTTCCCACTACATGCTGTTTGACGACATTGAGGCTATCGAAGTGATTGCTCGTTCAATTACCGTTGAGCAGTTCAAGGGATACTGCTTCGGTAACATCTTAAAGTACAGACTACGTGCTGGTAAGAAGTCAGAGTTAGCGTACTTAGAGAAAGACCTAGCGAAAGCAGACTTCTATAAAGactgtctcttatacacatctccgagccca                                                         >  1:75935/1‑273 (MQ=255)`  
                                                                                                                                                                                                                                                 `gATGGACCCACGTAAACCACATCTGAATAAACCTGAGGTAACTCATACAGATGACCAGCCTTCCGCTGAGACAATCGAAGGTGTCACTAAGCCTTCCCACTACATGCTGTTTGACGACATTGAGGCTATCGAAGTGATTGCTCGTTCAATGACCGTTGAGCAGTTCAAGGGATACTGCTTCGGTAACATCTTAAAGTACAGACTACGTGCTGGTAAGAAGTCAGAGTTAGCGTACTTAGAGAAAGACCTAGCGAAAGCAGACTTCTATAAAGAACTCTTTGAGAAACATAAGGATAAATGt                                                         <  1:712346/301‑1 (MQ=255)`  
                                                                                                                                                                                                                                                 `aagagacagaCGTAAACCACATCTGAATAAACCTGAGGTAACTCCTACAGATGACCAGCCTTCCGCTGAGACAATCGAAGGTGTCACTAAGCCTTCCCACTACATGCTGTTTGACGACATTGAGGCTATCGAAGTGATTGCTCGTTCAATGACCGTTGAGCAGTTCAAGTGATACTGCTTCGGTAACATCTTAAAGTACAGACTACGTGCTGGTAAGAAGTCAGAGTTAGCGTACTTAGAGAAAGACCTAGCGAAAGCAGACTTCTATAAAGAACTCTTTGAGAAACATAAGGATAAATGt                                                         <  1:778185/292‑1 (MQ=255)`  
                                                                                                                                                                                                                                                    `ggACCCACGTAAACCACATCTGAATAAACCTGAGGTAACTCCTACAGATGACCAGCCTTCCGCTGAGACAATCGAAGGTGTCACTAAGCCTTCCCACTACATGCTGTTTGACGACATTGAGGCTATCGAAGTGATTGCTCGTTCAATGACCGTTGAGCAGTTCAAGGGATACTGCTTCGGTAACATCTTAAAGTACAGACTACGTGCTGGTAAGAAGTCAGAGTTAGCGTACTTAGAGAAAGACCTAGCGAAAGCAGACTTCTATAAAGAACTCTTTGAGAAACATAAGGATAAATGTTAt                                                      >  1:701660/1‑301 (MQ=255)`  
                                                                                                                                                                                                                                                               `aaCCACATCTGAATAAACCTGAGGTAACTCCTACAGATGACCAGCCTTCCGCTGAGACAATCGAAGGTGTCACTAAGCCTTCCCACTACATGCTGTTTGACGACATTGAGGCTATCGAAGTGATTGCTCGTTCAATGACCGTTGAGCAGTTCAAGGGATACTGCTTCGGTAACATCTTAAAGTACAGACTACGTGCTGGTAAGAAGTCAGAGTTAGCGTACTTAGAGAAAGACCTAGCGAAAGCAGACTTCTATAAAGAACTCTTTGAGAAACATAAGGATAAATGTTATGCATAACTTCa                                           >  1:11317/1‑301 (MQ=255)`  
                                                                                                                                                                                                                                                               `aaCCACATCTGAATAAACCTGAGGTAACTCCTACAGATGACCAGCATTCCGCTGAGACAATCGAAGGTGTCACTAAGCCTTCCCACTACATGCTGTTTGACGACATTGAGGCTATCGAAGTGATTGCTCGTTCAATGACCGTTGAGCAGTTCAAGGGATACTGCTTCGGTAACATCTTAAAGTACAGACTACGTGCTGGTAAGAAGTCAGAGTTAGCGTACTTAGAGAAAGACCTAGCGAAAGCAGACTTCTATAAAGAACTCTTTGAGAAACATAAGGATAAATGTTATGcctgtctctt                                           >  1:142983/1‑292 (MQ=255)`  
                                                                                                                                                                                                                                                                    `caTCTTAATAAACCTGAGGTAACTCCTACAGATGACCAGCCTTCCGCTGAGACAATCGAAGGTGTCACTAAGCCTTCCCACTACATGCTGTTTGACGACATTGAGGCTATCTAAGTGATTGCTCGTTCAATGACCGTTGAGCAGTTCAAGGGATACTGCTTCGGTAACATCTTAAAGTACAGACTACGTGCTGGTAAGAAGTCAGAGTTAGCGTACTTAGAGAAAGACCTAGCGAAAGAAGACTTCTATAAAGAACTCTTTGAGAAACATAAGGAtctgtctcttatacacatctccgagc                                      >  1:468781/1‑276 (MQ=255)`  
                                                                                                                                                                                                                                                                       `gtataagagacagAGGTAACTCCTACAGATGACCAGACTTCCGCTGAGACAATCGAAGGTGTCACTAAGCCTTCCCACTACATGCTGATTGACGACATTGAGGCTATCGAAGTGATTGCGCGTTCAATGACCGTTGGGCAGTTCAAGGGATACTGCTGCGGTAACATCTTAAAGTACAGACTACGTGCTGGTAAGAAGTCAGAGTTAGCGTACTTAGAGAAAGACCTAGCGAAAGCAGACTTCTATAAAGAACTCTTTGAGAAACATAAGGATAAATGTTATGCATAACTTCAAGTCAAcc                                   <  1:35615/289‑1 (MQ=255)`  
                                                                                                                                                                                                                                                                       `cTGAATAAACCTGAGGTAACTCCTACAGATGACCAGCCTTCCGCTGAGACAATCGAAGGTGTCACTAAGCCTTCCCACTACATGCTGTTTGACGACATTGAGGCTATCGAAGTGATTGCTCGTTCAATGACCGTTGAGCAGTTCAAGGGATACTGCTTCGGTAACATCTTAAAGTACAGACTACGTGCTGGTAAGAAGTCAGAGTTAGCGTACTTAGAGAAAGACCTAGCGAAAGCAGACTTCTATAAAGAACTCTTTGAGAAACATAAGGATAAATGTTATGCATAACTTCAAGTCAAcc                                   >  1:544302/1‑301 (MQ=255)`  
                                                                                                                                                                                                                                                                        `ggagatgtgtataagagacagcTACAGATGACCAGCCTTCCGCTGAGACAATCGAAGGTGTCACTAAGCCTTCCAACTACATGCTGTTTGACGACATTGAGGCTATCGAAGTGATTGCTTGTTCAATGACCGTTGAGCAGTTCAAGGGATACTGCTTCGGTAACATCTTAAAGTACAGACTACGTGCTGGTAAGAAGTCAGAGTTAGCGTACTTATAGAAAGACCTAGCGAAAGCAGACTTCTATAAAGAACTCTTTGAGAAACATAAGGATAAATGTTATGCATAACTTCAAGTCAAccc                                  <  1:50924/280‑1 (MQ=255)`  
                                                                                                                                                                                                                                                                         `gAATAAACCTGAGGTAACTCCTACAGATGACCAGCCTTCCGCTGAGACAATCGAAGGTGTCACTAAGCCTTCCCACTACATGCTGTTTGATGACATTGAGGCTATCGAAGTGATTGCTCGTTCAATGACCGTTGAGCAGTTCAAGGGATACTGCTTCGGTAACATCTTAAAGTACAGACTACGTGCTGGTAAGAAGTCAGAGTTAGCGTACTTAGAGAAAGACCTAGCGAAAGCAGACTTCTATAAATAACTCTTTGAGAAACATAAGGATAAATGTTATGCATAACTTAAAGTCAAccca                                 >  1:92403/1‑300 (MQ=255)`  
                                                                                                                                                                                                                                                                         `gAATAAACCTGAGGTAACTCCTACAGATGACCAGCCTTCCGCTGAGACAATCGAAGGTGTCACTAAGCCTTCCCACTACATGCTGTTTGACGACATTGAGGCTATCGAAGTGATTGCTCGTTCAATGACCGTTGAGCATTTCAAGGGATACTGCTTCGGTAACATCTTAAAGTACAGACTACGTGCTGGTAAGAAGTCAGAGTTAGCGTACTTAGAGAAAGACCTAGCGAAAGCAGACTTCTATAAAGAACTCTTTGAGGAACATAAGGATAAATGTTATGCATAACTTCAAGTCAAcccc                                 <  1:271222/301‑1 (MQ=255)`  
                                                                                                                                                                                                                                                                          `gggtgtatagcttgcttagagaaaGATGACCAGCCTTCCGCTGAGACAATCGAAGGTGTCACTAAGCCTTCCCACTACATGCTGTTTGACGACATTGAGGCTATCGAAGTGATTGCTCGTT‑AATGACCGTTGAGCAGTTCAAGGGATACTGCTTCGGTAACATCTTAAAGTACAGACTACGTGCTGGTAAGAAGTCAGAGTTAGCGTACTTAGAGAAAGACCTAGCGAAAGCAGACTTCTATAAAGAACTCTTTGAGAAACATAAGGATAAATGTTATGCATAACTTCAAGTCAACCCCAc                               >  1:409290/24‑301 (MQ=255)`  
                                                                                                                                                                                                                                                                            `aaaataaaagaaagagatgaaagaagaACAGCCTTCCGCTGAGACAAACGAAGGAGTAACTAAGCCTTACAAATAAAAGCTGTTTGAAAAAAATGAGGCAAACGAAGTGATGGATCGTTCAAAGACAGTTGAGCAGGTCAAGGGATAATGATTAGGGAAAATAGTAAAGAACAGAATAAGTAAAAGTAAGAAGTCAGAGAAAGGGTACTTAGAGAAAGAACGAGAGAAAGCAGAATGCTATAAAGAACTATTTGAGAAACATAAGGATAAATGTTATGCATAACTTCAAGTCAACCCCAcc                              <  1:42742/280‑1 (MQ=255)`  
                                                                                                                                                                                                                                                                                   `gAGGTAACTCCTACAGATGACCAGCCTTCCGCTGAGACAATCGAAGGTGTCACTAAGCCTTCCAACTACATGCTGTTTGACGACATTGAGGCTATCGAAGTGATTGCTCGTTCAATGACCGTTGAGCAGTTCAAGGGATACTGCTTCGGTAACATCTTAAAGTACAGACTACGTGCTGGTAAGAAGTCAGAGTTAGCGTACTTAGAGAAAGACCTAGCGAAAGCAGACTTCTATAAAGAACTCTTTGAGAAACATAAGGATAAATGTTATGCATAACTTCAAGTCAACCCCACCTGCCGAc                       >  1:730012/1‑301 (MQ=255)`  
                                                                                                                                                                                                                                                                                      `ggagatgtgtataagagacAGCCTTCCGCTGAGACAATCGAAGGTGTCACTAAGCCTTCCCACTACATGCTGTTTGACGACATTGAGGCTATCGAAGTGATTGCTCGTTCAATGACCGTTGAGCAGTTCAAGGGATACTGCTTCGGTAACATCTTAAAGTACAGACTACGTGCTGGTAAGAAGTCAGAGTTAGCGTACTTAGAGAAAGACCTAGCGAAAGCAGACTTCTATAAAGAACTCTTTGAGAAACATAAGGATAAATGTTATGCATAACTTCAAGTCAACCCCACCTGCCGACAGc                    <  1:127272/283‑1 (MQ=255)`  
                                                                                                                                                                                                                                                                                      `gTAACTCCTACAGATTACCAGCCTTCCGCTTAGACAATCGAAGGTGTCACTAAGCCTTCCCACTACATGCTGTTTGACGACATTGAGGCTATCGAAGTGATTGCTCGTTCAATGACCGTTGAGCAGTTCAAGGGATACTGCTTCGGTAACATCTTAAAGTACATACTACGTGCTGGTAAGAAGTCAGAGTTAGCGTACTTAGAGAAAGACCTAGCGAAAGCAGACTTCTATAAAGAACTCTTTGAGAAACATAAGGATAAATGTTATGCATAACTTCAAGTCAACCCCACCTGCCGACAGc                    <  1:316638/301‑1 (MQ=255)`  
                                                                                                                                                                                                                                                                                       `tAACTCCTACAGATGACCAGCCTTCCGCTGAGACAATCGAAGGTGTCACTAAGCCTTCCCACTACATGCTGTTTGACGACATTTAGGCTATCGAAGTGATTGCTCGTTCAATGACCGTTGAGCAGTTCAAGGGATACTGCTTCGGTAACATCTTAAAGTACAGACTACGTGCTGGTAAGAAGTCAGAGTTATCGTACTTAGAGAAAGACCTAGCGAAAGCAGACTTCTATAAAGAACTCTTTGAGAAACATAAGGATAAATGTTATGCATAACTTCAAGTCAACCCCACCTGCCGACAGcc                   <  1:235545/301‑1 (MQ=255)`  
                                                                                                                                                                                                                                                                                       `tAACTCCTACAGATGACCAGCCTTCCGCTGAGACAATCGAAGGTGTCACTAAGCCTTCCCACTACATGCTGTTTGACGACATTGAGGCTATCGAAGTGATTGCTCGTTCAATGACCGTTGAGCAGTTCAAGGGATACTGCTTCGTTAACATCTTAAAGTACAGACTACGTGCTGGTAAGAAGTCAGAGTTAGCGTACTTAGAGAAAGACCTAGCGAAAGCAGACTTCTATAAAGAACTCTTTTAGAAACATAAGGATAAATGTTATGCATAACTTCAAGTCAACCCCACCTGCCGACAGcc                   <  1:686128/301‑1 (MQ=255)`  
                                                                                                                                                                                                                                                                                           `tCCTACAGATGACCAGCCTTACGCTGAGACAATCGAAGGTGTAACTAAGCCTTCCCGCTACATGCTGTTTGACGACATTGAGGCTATCGAAGTGATTGCTCGTTCAATGACCGTTGAGCAGTTCAAGGGATACTGCTTCGGTAACATCTTAAAGTACAGACTACGTGCTGGTAAGAAGTCAGAGTTAGCGTACTTAGAGAAAGACCTAGCGAAAGCAGACTTCTATAAAGAACTCTTTGAGAAACATAAGGATAAATGTTATGCATAACTTCAAGTCAACCCCACCTGCCGACAGCCTATc               <  1:402586/301‑1 (MQ=255)`  
                                                                                                                                                                                                                                                                                            `ccTACAGATGACCAGCCTTCCGCTGAGACAATCGAAGGTGTCACTAAGCCTTCCCACTACATGCTGTTTGACGACATTGAGGCTATCGAAGTGATTGCTCGTTCAATGACCGTTGAGCAGTTCAAGGGATACTGCTTCGGTAACATCTTAAAGTACAGACTACGTGCTGGTAAGAAGTCAGAGTTAGCGTACTTAGAGAAAGACCTAGCGAATGCAGACTTCTATAAAAAACTCTTTGATAAACCTAAAGATCAATGTTATGCATAACTTCAAGTCAACCACACCTGCCGACAGCCTATCt              >  1:81417/1‑301 (MQ=255)`  
                                                                                                                                                                                                                                                                                             `cTACAGATGACCAGCCTTCCGCTGAGACAATCGAAGGTGTCACTAAGCCTTCCCACTACATGCTGTTTGACGACATTGAGGCTATCGAAGTGATTGCTCGTTCAATGACCGTTGAGCAGTTCAAGGGATACTGCTTCGGTAACATCTTAAAGTACAGACTACGTGCTGGTAAGAAGTCAGAGTTAGCGTACTTAGAGAAAGACCTAGCGAAAGCAGACTTCTATAAAGAACTCTTTGAGAAACATAAGGATAAATGTTATGCATAACTTCAAGTCAACCCCACCTGCCGACAGCCTATCtg             <  1:297831/301‑1 (MQ=255)`  
                                                                                                                                                                                                                                                                                             `cTACAGATGACCAGCCTTCCGCTGAGACAATCGAAGGTGTCACTAAGCCTTCCCACTACATGCTGTTTGACGACATTGAGGCTATCGAAGTGATTGCTCGTTCAATGACCGTTGAGCAGTTCAAGGGATACTGCTTCGGTAACATCTTAAAGTACAGACTACGTGCTGGTAAGAAGTCAGAGTTAGCGTACTTAGAGAAAGACCTAGCGAAAGCAGACTTCTATAAAGAACTCTTTGAGAAACATAAGGATAAATGTTATGCATAACTTCAAGTCAACCCCACCTGCAGACAGCCTATCtg             <  1:765128/301‑1 (MQ=255)`  
                                                                                                                                                                                                                                                                                                `cAGATGACCAGCCTTCCGCTGAGACAATCGAAGGTGTCACTAAGCCTTCCCACTACATGCTGTTTGACGACATTGATGCTATCGAAGTGATTGCTCGTTCAATGACCGTTTAGCAGTTCAAGGGATACTGCTTCGGTAACATCTTAAAGTACAGACTACGTGCTGGTAAGAAGTCAGAGTTAGCGTACTTATAGAAAGACCTAGCGAAAGCAGACTTCTATAAAGAACTCTTTGAGAAACATAAGGATAAATGTTATGCATAACTTCAAGTCAACCCCACCTGCCGACAGCCTATCtgatg          <  1:74771/301‑1 (MQ=255)`  
                                                                                                                                                                                                                                                                                                     `gACCAGCCTTCCGCTGAGACAATCGAAGGTGTCACTAAGCCTTCCCACTACATGCTGTTTGACGACATTGAGGCTATCGAAGTGATTGCTCGTTCAATGACTGTTGAGCAGTTCAAGGGATACTGCTTCGGTAACATCTTAAAGTACAGACTACGTGCTGGTAAGAATTCAGAGTTAGCGTACTTAGAGAAAGACCTAGCGAAAGCAGACTTCTATAAAGAACTCTTTGAGAAACATAAGGATAAATGTTATGCATAACTTCAAGTCAACCCCACCTGCCGACAGCCTATCTGATGACTTc     >  1:454106/1‑301 (MQ=255)`  
                                                                                                                                                                                                                                                                                                     `gACCAGCCTTCCGCTGAGACAATCGAAGGTGTCACTAAGCCTTCCCACTACATGCTGTTTGACGACATTGAGGCTATCGAAGTGATTGCTCGTTCAATGACCGTTGAGCAGTTCAAGGGATACTGCTTCGGTAACATCTTAAAGTACAGACTACGTGCTGGTAAGAAGTCAGAGTTAGCGTACTTAGAGAAAGACCTAGCGAAAGCAGACTTCTATAAAGAACTCTTTGAGAAACATAAGGATAAATGTTATGCATAACTTCAAGTCAACCCCACCTGCCGACAGCCTATCTGATGACTTc     >  1:396861/1‑301 (MQ=255)`  
                                                                                                                                                                                                                                                                                                       `ccAGCCTTCCGCTGAGACAATCGAAGGTGTCACTAAGCCTTCCCACTACATGCTGTTTGACGACATTGAGGCTATCGAAGTGATTGCTCGTTCAATGACCGTTGAGCAGTTCAAGGGATACTGCTTCGGTATCATCTTAAAGTACAGACTACGTGCTGGTAAGAAGTCAGAGTTAGCGTACTTAGAGAAAGACCTAGCGAAAGCAGACTTCTATAAAGAACTCTTTGAGAAACATAAGGATAAATGTTATGCATAACTTCAAGTCAACCCCACCTGCCAACAGCCTATCTGATGACTTcac   >  1:577490/1‑301 (MQ=255)`  

                                                                                                                                                                                                                                                                                                         `|`                                                                                                                                                                                                                                                                                                             
`CGCCAGTACAAGCAACTGGGTGTATAGCTTGCTTAGAGAAAGATGACCGTTATCCACACACCTGTAACAAAGGAGCTAACGATATGACCGAACGTGAACAAGAGATGATCATTAAGTTGATAGACAATAATGAAGGTCGCCCAGATGATTTGAATGGCTGCGGTATTCTCTGCTCCAATGTCCCTTGCCACCTCTGCCCCGCAAATAACGATCAAAAGATAACCTTAGGTGAAATCCGAGCGATGGACCCACGTAAACCACATCTGAATAAACCTGAGGTAACTCCTACAGATGACCAGCCTTCCGCTGAGACAATCGAAGGTGTCACTAAGCCTTCCCACTACATGCTGTTTGACGACATTGAGGCTATCGAAGTGATTGCTCGTTCAATGACCGTTGAGCAGTTCAAGGGATACTGCTTCGGTAACATCTTAAAGTACAGACTACGTGCTGGTAAGAAGTCAGAGTTAGCGTACTTAGAGAAAGACCTAGCGAAAGCAGACTTCTATAAAGAACTCTTTGAGAAACATAAGGATAAATGTTATGCATAACTTCAAGTCAACCCCACCTGCCGACAGCCTATCTGATGACTTCACA  >  NC_001604.1/8206‑8802`

  

Alignment Legend

Aligned base mismatch/match (shaded by quality score): `ATCG/ATCG` `< 3 ≤` `ATCG/ATCG` `< 16 ≤` `ATCG/ATCG` `< 28 ≤` `ATCG/ATCG` `< 37 ≤` `ATCG/ATCG` `< 38 ≤` `ATCG/ATCG`

Unaligned base: `atcg`    Masked matching base: `atcg`    Alignment gap: `‑`    Deleted base: `‑`

Reads not counted as support for junction

read\_name Not counted due to insufficient overlap past the breakpoint.

read\_name Not counted due to not crossing MOB target site duplication.
